# Versorgung von Choanalatresien in Deutschland: Dorsale Septumresektion und Stents im Fokus

**DOI:** 10.1007/s00106-023-01410-x

**Published:** 2024-01-08

**Authors:** Miray-Su Yılmaz Topçuoğlu, Antje Hammitsch-Mayer, Peter K. Plinkert, Ingo Baumann

**Affiliations:** 1 Hals‑, Nasen- und Ohrenklinik, Universitätsklinik Heidelberg, Im Neuenheimer Feld 400, 69120 Heidelberg, Deutschland; 2 Praxis für Gynäkologie und Geburtshilfe Dr. med. Ulrike Steinhoff, Berlin, Deutschland

**Keywords:** Pädiatrische Rhinologie, Septumresektion, Stents, Versorgungssituation, Nasenchirurgie, Pediatric rhinology, Septal resection, Stents, Supply situation, Nasal surgery

## Abstract

**Hintergrund:**

Verschiedene chirurgische Techniken mit transpalatinalen, transseptalen und transnasalen Zugängen zur operativen Therapie der Choanalatresie wurden in den letzten 200 Jahren entwickelt. Die endoskopische endonasale Chirurgie mit dorsaler Septumresektion und ohne Stents ist der aktuelle wissenschaftliche Trend, da so hohe Erfolgsraten mit geringen Komplikationsraten erreicht werden können. Diese Studie untersuchte, ob sich diese Technik tatsächlich flächendeckend in Deutschland durchgesetzt hat und welche Rolle die Anwendung von Stents hierbei spielt.

**Methoden:**

Insgesamt 52 Hals-Nasen-Ohren(HNO)-Kliniken in Deutschland, davon alle 39 HNO-Universitätskliniken und 13 nicht-universitäre HNO-Kliniken der Maximalversorgung, wurden befragt, welche chirurgische Technik sie für die Choanalatresieresektion verwenden und ob Stents zum Einsatz kommen.

**Ergebnisse:**

Für die dorsale Septumresektion gaben 39 von 44 antwortenden Kliniken (89 %) an, dorsale Septumteile zu resezieren. Bei den Universitätskliniken lag der Anteil bei 85 %, bei den nicht-universitären Kliniken bei 100 %.

Für die Anwendung von Stents gaben 20 von 48 antwortenden Kliniken (42 %) an, keine Stents zu verwenden. Bei den Universitätskliniken lag der Anteil bei 39 %, bei den nicht-universitären Kliniken bei 50 %.

**Schlussfolgerung:**

Die endoskopische endonasale Choanalatresieresektion mit dorsaler Septumresektion wird in den großen HNO-Kliniken Deutschlands größtenteils als Standardtechnik verwendet. Die routinemäßige Verwendung von Stents ist noch weit verbreitet. Die Reduktion der Stentnutzung in der Choanalatresieversorgung und die Verwendung dieser Methode nur in schwierigen Einzelfällen sollte künftiges Ziel sein.

## Abstract (en)

**Background:**

Various surgical techniques using transpalatinal, transseptal, and transnasal approaches for surgical repair of choanal atresia have been developed over the past 200 years. Endoscopic endonasal surgery with resection of dorsal septal parts and without the use of stents is the current scientific trend, as high success rates with low complication rates can be achieved. This study examined whether this technique has actually become standard in Germany, and also investigates the role played by stents.

**Methods:**

A total of 52 ear, nose, and throat (ENT) hospitals in Germany, including all 39 university ENT hospitals and 13 non-university maximum-care ENT hospitals, were asked which surgical technique they use for choanal atresia repair and whether stents are used.

**Results:**

For dorsal septal resection, 39 of 44 responding hospitals (89%) indicated that they resect dorsal septal parts, 85% of the university hospitals and 100% of the non-university hospitals. For use of stents, 20 of 48 responding hospitals (42%) reported not using stents, whereas 39% of the university hospitals and 50% of the non-university hospitals do use them.

**Conclusion:**

Endoscopic endonasal choanal atresia repair with resection of dorsal septal parts is, in most instances, used as the standard technique in large ENT hospitals in Germany. Routine use of stents is still widespread. The future aim should be to reduce the use of stents in the treatment of choanal atresia and to use this method only in difficult, individual cases.

Die Choanalatresie ist selten und stellt in der pädiatrischen Rhinologie eine herausfordernde Erkrankung dar, zu deren Therapie Expertise benötigt wird. Seit über 200 Jahren ist die Therapie der Choanalatresie Thema in der Wissenschaft. Verschiedene chirurgische Techniken wurden erprobt und entwickelt. Der aktuelle Trend geht hin zur endoskopischen endonasalen Choanalatresieresektion mit dorsaler Septumresektion und weg von Stents mit dem Ziel der Reduktion des Komplikations- und Rezidivrisikos. Die aktuelle Versorgungssituation in Deutschland ist das Thema der vorliegenden Untersuchung.

## Hintergrund und Fragestellung

**Figure 1:**
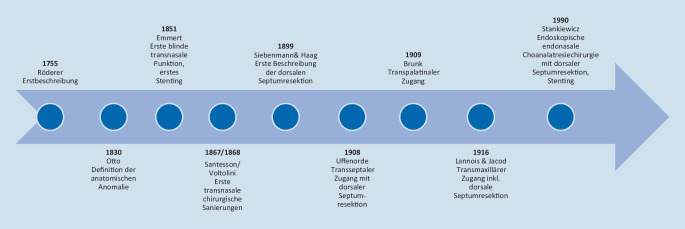

Die angeborene Choanalatresie stellt eine seltene und herausfordernde Erkrankung der pädiatrischen Rhinologie dar und wird chirurgisch therapiert [4, 19]. Die Choanalatresie wird im Fall eines beidseitigen Vorhandenseins notfallmäßig bereits kurz nach der Geburt und elektiv im Fall einer einseitigen Choanalatresie in Abhängigkeit von den störenden Symptomen operiert [4, 18, 19]. Da im Rahmen von chirurgischen Eingriffen in der pädiatrischen Rhinologie besonders die nasalen Wachstumszonen geschont werden müssen, wurden seit über 200 Jahren zahlreiche Veröffentlichungen zu diesem Thema verfasst und die Erfahrungen diskutiert [5, 21]. Die chirurgischen Techniken wurden den zeitgemäßen Gegebenheiten Schritt für Schritt angepasst (Abb. 1; [4, 5, 19–21]). Nach der ersten Beschreibung der Choanalatresie 1755 durch Johann Georg Röderer [4, 20] und Adolf Wilhelm Otto 1830 [15, 20] führte Carl Emmert 1851 die erste blinde transnasale Choanalatresiepunktion mittels Trokar durch [10, 15]. Weitere transnasale Choanalatresieoperationen mit punktierender Technik folgten [15]. Im Jahr 1899 beschrieben Siebenmann und Haag erstmalig die dorsale Septumresektion zur Verhinderung einer Restenose [15]. Uffenorde publizierte 1908 einen neuartigen transseptalen Zugang mit großzügigen Septumresektionen, unter anderem auch der zuvor von Siebenmann und Haag beschriebenen dorsalen Septumanteile [15, 23]. Diese Technik führte zwar zu einer stabilen Neochoane, jedoch auch zu Wachstumsstörungen des Gesichts und der Nase [15, 23]. Ab 1909 wurde zunehmend die transpalatinale Choanalatresieresektion durchgeführt, welche neben der transnasalen Technik lange Zeit führende Methode war [5, 14, 15]. Mit dem allmählichen Beginn der endoskopischen Nasenchirurgie ab der zweiten Hälfte des 20. Jahrhunderts und der Einführung der endoskopischen endonasalen Choanalatresieoperation in den 1980er- und 1990er-Jahren änderte sich die chirurgische Technik der Choanalatresieresektion grundlegend [5]. Stankiewicz führte mit seiner Veröffentlichung im Januar 1990 erstmalig die endoskopisch endonasale Choanalatresieresektion mit dorsaler Septumteilresektion als chirurgische Technik ein und verwendete postoperativ die auch bereits zuvor bekannten Stents [20]. Die Technik der dorsalen Septumresektion hat sich über die letzten 30 Jahre zunehmend als grundlegende chirurgische Technik durchgesetzt [5, 9, 14, 21]. Die Verwendung von Stents dagegen, welche initial standardmäßig postoperativ verwendet wurden, wird aufgrund zahlreicher Komplikationen und dem erhöhten Rezidivrisiko, welches die Stents mit sich bringen, zunehmend kontrovers diskutiert [1–3, 5, 7, 11, 12, 22]. Strychowsky et al. zeigten in einer Metaanalyse auf, dass die chirurgischen Erfolgsraten vergleichbar waren zwischen Patientengruppen, bei denen Stents verwendet wurden, und solchen, bei denen keine Stents verwendet wurden. Sie zeigten jedoch auch auf, dass die Nutzung von Stents mit mehr postoperativen Komplikationen assoziiert ist [22]. Neben der optimierten chirurgischen Technik spielt auch eine intensive postoperative Nasenpflege eine essenzielle Rolle für das Outcome [4, 19]. In Deutschland führen zahlreiche Hals-Nasen-Ohren(HNO)-Kliniken im universitären und nicht-universitären Bereich Resektionen von Choanalatresien durch. Die eigene klinische Erfahrung des letzten Jahrzehnts mit Revisionsoperationen von initial extern operierten Patienten zeigte, dass in diesen Fällen die dorsale Septumresektion oftmals nicht oder nicht ausreichend erfolgte. Anamnestisch war es bei diesen Patienten typisch, dass die nasale Obstruktion schon einige Tage oder Wochen nach der Operation bzw. nach der Entfernung der Stents wieder relevant war. Daher war das Ziel dieser Studie zu untersuchen,

1. ob und inwieweit sich die dorsale Septumresektion bei der Versorgung von kongenitalen Choanalatresien in Deutschland etabliert hat und
2. wie verbreitet die Anwendung von Stents tatsächlich ist.

## Methoden

Insgesamt wurden deutschlandweit 52 Kliniken für HNO-Heilkunde bezüglich ihrer Methode bei der Choanalatresieresektion und zur Verwendung von Stents im Rahmen dieser Operation befragt. Es wurden alle 39 deutschen Universitätskliniken für HNO-Heilkunde und 13 nicht-universitäre HNO-Kliniken der Maximalversorgung eingeschlossen. Die Kliniken wurden per E‑Mail kontaktiert. Dabei sollten die Kliniken ihre chirurgischen Techniken angeben, insbesondere, ob sie standardmäßig dorsale Septumteile resezierten. Zudem wurde gefragt, ob Stents zur Verwendung kommen. Die Beantwortung der Fragen durch die Kliniken erfolgte größtenteils per E‑Mail, teilweise auch telefonisch.

## Ergebnisse

### Dorsale Septumresektion

Von insgesamt 52 befragten Kliniken (34 Universitätskliniken, 10 nicht-universitäre Kliniken der Maximalversorgung) äußerten sich 44 zur chirurgischen Technik. Es gaben 39 Kliniken (89 %) an, dorsale Septumteile zu resezieren. Bei den Universitätskliniken lag der Anteil bei 85 % (29/34 Kliniken), bei den nicht-universitären Kliniken lag der Anteil bei 100 % (10/10 Kliniken). Die Tab. 1 stellt diese Daten im Detail dar.

**Table 1:** 

Dorsale Septumresektion	*n*	Ja	Nein	N/A
Gesamt	44/52	39 (89 %)	5 (11 %)	8
Universitätskliniken	34/39	29 (85 %)	5 (15 %)	5
Nicht-universitäre Kliniken	10/13	10 (100 %)	0 (0 %)	3

*n* Anzahl der antwortenden Kliniken/Anzahl der insgesamt befragten Kliniken. *Ja/Nein* Angaben in den Spalten „Ja“ und „Nein“ als absolute Zahlen (Prozentzahlen). *N/A* Anzahl von Kliniken, die auf die Befragung keine Antworten gaben

### Nutzung von Stents

Von insgesamt 48 antwortenden Kliniken (36 Universitätskliniken, 12 nicht-universitäre Kliniken der Maximalversorgung) gaben 20 Kliniken (42 %) an, keine Stents zu verwenden. Von diesen Kliniken verwenden 21/48 Kliniken (44 %) routinemäßig Stents und 7/48 (15 %) verwenden fallabhängig Stents.

Bei den Universitätskliniken gaben 14/36 Kliniken (39 %) an, keine Stents zu verwenden. Von diesen Universitätskliniken verwenden 16/36 Universitätskliniken (44 %) routinemäßig Stents und 6/36 Universitätskliniken (17 %) verwenden nur fallabhängig Stents.

Bei den nicht-universitären Kliniken der Maximalversorgung gaben 6/12 Kliniken (50 %) an, keine Stents zu verwenden. Von diesen nicht-universitären Kliniken verwenden 5/12 Kliniken (42 %) routinemäßig Stents und 1/12 Kliniken (8 %) verwendet nur fallabhängig Stents. Die genauen Daten können der Tab. 2 entnommen werden.

**Table 2:** 

Stents	*n*	Ja	Nein	Fallabhängig	N/A
Gesamt	48/52	21 (44 %)	20 (42 %)	7 (15 %)	4
Universitätskliniken	36/39	16 (44 %)	14 (39 %)	6 (17 %)	3
Nicht-universitäre Kliniken	12/13	5 (42 %)	6 (50 %)	1 (8 %)	1

*n* Anzahl der antwortenden Kliniken/Anzahl der insgesamt befragten Kliniken. *Ja/Nein* Angaben in den Spalten „Ja“ und „Nein“ als absolute Zahlen (Prozentzahlen). *N/A* Anzahl von Kliniken, die auf die Befragung keine Antworten gaben

## Diskussion

Mit einer Inzidenz von 1:5000 bis 8000 Geburten ist die Choanalatresie zwar selten [6, 14, 24], aber dennoch so häufig, dass eine chirurgische Versorgung in betreuenden HNO-Kliniken keine Rarität darstellt. Auch wenn die Therapie jedes einzelnen Patienten bezüglich des Zeitpunkts der Operation und des chirurgischen Vorgehens individuell geplant werden muss, sollte dennoch ein standardisiertes Verfahren zur Qualitäts- und Erfolgssicherung angewandt werden.

Der erste Fokus der Studie lag auf der dorsalen Septumresektion. Dass die dorsale Septumresektion eine hervorragende Technik für die erfolgreiche Therapie der Choanalatresie darstellt, wurde bereits 1899 erkannt, hatte sich jedoch lange Zeit noch nicht als alleinige Standardtechnik durchgesetzt und häufig Wachstumsstörungen des Mittelgesichts zur Folge [15]. Im 19. Jahrhundert wurden zudem zahlreiche transpalatinale Choanalatresieresektionen durchgeführt [15]. Mit Beginn der endoskopischen Nasenchirurgie seit Anfang 1990 ist die dorsale Septumresektion zunehmend der chirurgische Standard, da so kontrolliert dorsale Septumteile entfernt werden können [20].

Im Vergleich zu transseptalen und transpalatinalen Zugangswegen [16–18, 24] stellt die endoskopisch endonasale Choanalatresieresektion mit dorsaler Septumresektion inzwischen die Methode der Wahl dar, da die Komplikations- und Rezidivraten vergleichsweise gering sind [5, 9, 14]. Es stellte sich die Frage, inwieweit sich die dorsale Septumresektion in Deutschland auch tatsächlich als Standardtechnik etabliert hat, da eigene klinische Erfahrungen zeigten, dass dorsale Septumteile bei Patienten, die eine Revisionsoperation benötigten, zum größten Teil nur unzureichend reseziert waren.

Unsere Studie konnte zeigen, dass die dorsale Septumresektion in der weit überwiegenden Anzahl der befragten Kliniken als grundlegende Operationstechnik etabliert ist. Allerdings wird durch die Befragung auch deutlich, dass in der Behandlung dieses seltenen Krankheitsbildes noch Weiterbildungsbedarf besteht, sowohl für das grundlegende theoretische Verständnis als auch bezüglich der operativen Möglichkeiten. Da eine spezielle Weiterbildung in pädiatrischer HNO-Heilkunde anders als in vielen anderen Ländern in Deutschland nicht existiert, müssen andere Wege für eine Förderung dieser Wissensvermittlung und die diesbezügliche chirurgische Ausbildung gefunden werden.

Der zweite Fokus der Studie war die Untersuchung der Rolle von Stents in der postoperativen Phase. Die Nutzung von Stents wurde bereits in zahlreichen Publikationen kontrovers diskutiert. Von vielen Autoren werden Stents aufgrund des erhöhten Komplikations- und Rezidivrisikos sehr kritisch gesehen [5, 7, 8, 13, 18, 22]. Im Gespräch mit betroffenen Familien zeigt sich die Situation insbesondere bei langfristiger Stenteinlage problematisch. Diese bedeutet für die Familien oftmals eine lange Krankenhausliegedauer, einen hohen pflegerischen Aufwand bis hin zur Notwendigkeit von 24-Stunden-Pflegediensten im häuslichen Umfeld und somit eine enorme Belastung für das Familienleben und die Patienten. Diese Studie zeigte, dass Stents bei 44 % der befragten Kliniken noch standardmäßig eingesetzt werden und zudem bei 8–17 % der Kliniken fallabhängig zum Einsatz kommen. Diesbezüglich scheint der Weiterbildungsbedarf in Deutschland höher zu sein als im Vergleich zur Operationstechnik bezüglich der dorsalen Septumresektion. Das Ziel sollte die Reservierung der Verwendung von Stents z. B. für syndromale Patienten mit komplexen anatomischen Situationen sein. Die Intensivierung des wissenschaftlichen und klinischen Austauschs ist erforderlich, um hier eine Änderung des Status quo zu erreichen.

Wege der zusätzlichen Wissensvermittlung könnten beispielswiese die Bildung von überregionalen Qualitätszirkeln zum Thema Management und Therapien seltener HNO-Erkrankungen sein, die Etablierung fester Standards in Form einer deutschen Leitlinie zur Choanalatresie, die Verankerung von Wissensinhalten zum Management seltener HNO-Erkrankungen auch im Weiterbildungskatalog und letztlich auch, den betroffenen Familien bzw. Selbsthilfegruppen auf Fachkongressen oder in Form von Artikeln verstärkt eine Stimme zu geben.

### Limitationen

In dieser Studie wurden 52 große Kliniken von insgesamt über 150 deutsche Kliniken mit HNO-Hauptabteilung befragt, sodass kein vollständiges Bild der Versorgungssituation in Deutschland erstellt werden kann. 44 teilnehmende Kliniken äußerten sich zur dorsalen Septumresektion, 48 Kliniken beantworteten die Frage nach der Verwendung von Stents. Grund für diesen Unterschied in der Antwortquote ist, dass sich vier der insgesamt 48 antwortenden Kliniken nicht zu dem Aspekt der Septumresektion äußerten. Eine weitere Einschränkung der Studie ist das Risiko der Interviewer- und Response-Bias, sodass Antworten aufgrund der Befragungssituation gegebenenfalls verfälscht wurden. Der Ad-hoc-Befragung sollte eine standardisierte doppelblinde Befragung folgen, die das anonymisierte Beantworten der Fragen ermöglicht und weitere Details wie zum Beispiel die Liegedauer und das Material der Stents sowie das genaue Nachsorgekonzept, welches für den Therapieerfolg essenziell ist, erhebt.

## Fazit für die Praxis

- Die endoskopische endonasale Choanalatresieoperation mit dorsaler Septumresektion ist auf einem guten Weg, sich als Goldstandardtechnik zu etablieren.
- Es bedarf jedoch noch weiterer Bemühungen, dies auch wirklich flächendeckend in ganz Deutschland zu gewährleisten.
- Stents werden noch in vielen Kliniken routinemäßig als Standard in der postoperativen Phase genutzt.
- Es besteht in Deutschland weiterhin der Bedarf, die Nutzung von Stents auf ein Minimum zu reduzieren und diese nur noch in ausgewählten Einzelfällen zu verwenden.
- Eine Intensivierung des wissenschaftlichen und klinischen Austauschs zur Therapie von kongenitalen Choanalatresien und der diesbezüglichen chirurgischen Ausbildung ist erforderlich, um eine Änderung des Status quo in Deutschland zu erreichen.
